# Influence of miniscrew-assisted rapid palatal expansion (MARPE) on the interdental papilla height of maxillary central incisors

**DOI:** 10.1007/s00784-023-05214-9

**Published:** 2023-08-19

**Authors:** Hao Chen, Aldin Kapetanović, Zhengguo Piao, Tong Xi, Jan G. J. H. Schols

**Affiliations:** 1 Department of Oral and Maxillofacial Surgery, Affiliated Stomatology Hospital of Guangzhou Medical University, Guangzhou, Guangdong China; 2 Department of Orthodontics and Craniofacial Biology, Radboud Institute for Health Sciences, Radboud University Medical Center, Nijmegen, The Netherlands; 3 Department of Oral and Maxillofacial Surgery, Radboud Institute for Health Sciences, Radboud University Medical Center, Geert Grooteplein 10, 6500 HB Nijmegen, The Netherlands

**Keywords:** Papilla recession, Miniscrew-assisted rapid palatal expansion (MARPE), Jemt classification, Bone crest, Crown morphology

## Abstract

**Objectives:**

To evaluate the influence of miniscrew-assisted rapid palatal expansion (MARPE) on the interdental papilla height of maxillary central incisors.

**Materials and methods:**

Patients who completed MARPE treatment at the Radboud University Medical Center between 2018 and 2021 were included in this retrospective study. The papilla height between the maxillary central incisors was evaluated on frontal intraoral photographs taken before expansion (T0) and 1.5 years after MARPE treatment (T1) using the Jemt classification. The difference in Jemt score at T0 and T1 was the primary outcome variable. In addition, gender, age, Angle classification, MARPE duration, midpalatal suture maturation stage, maximal central diastema (MCD) immediately after expansion, crown width to length ratio (W/L), pretreatment overlap of maxillary central incisors, and the distance between the approximal contact point of the central incisors and the bone crest (CP-B) were also record.

**Results:**

Twenty-two patients were included (2 men, 20 women, mean age 27.3 ± 8.8 years) and 4 patients (18%) showed a significant reduction in the Jemt score following MARPE (*p* = 0.04), indicating papilla recession. Interdental papilla recession was significantly associated with the increase of CP-B (*p* = 0.02), smaller W/L (*p* < 0.01), overlapping of maxillary central incisors (*p* < 0.01), and smaller MCD immediately after expansion (*p* = 0.02).

**Conclusions:**

One and a half years after MARPE, 18% of patients exhibited mild recession of papilla height of the maxillary central incisors. Overlapping and smaller W/L of maxillary central incisors were prognostic factors for interdental papilla recession.

**Clinical relevance:**

Clinicians have to be aware of and inform the patients about the occurrence of papilla recession following MARPE.

## Introduction

Maxillary dental arch expansion for orthodontic treatment is often associated with papilla recession [1, 2]. Noverraz et al. demonstrated that in one third of patients who underwent orthodontic treatment including surgically assisted rapid maxillary expansion (SARME), papilla recession between the maxillary central incisors occurred [3]. The recession of the interdental papilla is undesired as it negatively affects esthetics and increases food impaction [4]. The recession of the papilla between maxillary central incisors in particular leads to the presence of an esthetically displeasing “black triangle,” which is difficult to treat despite the use of periodontal regeneration techniques [5, 6]. Both patients and clinicians are increasingly concerned about the aesthetics of the papilla [7, 8].

Miniscrew-assisted rapid palatal expansion (MARPE) is a non-surgical treatment for transverse maxillary deficiency and is increasingly becoming the alternative to SARME to enlarge the transverse dimension of the maxillary arch [8–11]. MARPE is an orthodontic device with four miniscrews anchored into the palate delivering the expansion force directly to the maxilla in order to achieve skeletal expansion, with recent studies reporting that expansion was successful in 79.53 to 94.1% of cases [11, 12].

During MARPE treatment, a diastema between the maxillary central incisors gradually increases following the separation of the midpalatal suture. The alveolar bone crest between the central incisors is temporarily separated and the approximal contact point between the central incisors is temporarily lost. The crestal bone height and the presence of an approximal contact point are periodontically associated with papilla recession [13, 14]. Despite the absence of surgical trauma when using MARPE compared to SARME, the abovementioned effects of MARPE could potentially be detrimental to the preservation of the central papilla.

This study aimed to evaluate the influence of MARPE on the interdental papilla height of the maxillary central incisors and to assess potential predictors associated with the occurrence of papilla recession.

## Materials and methods

### Study design and setting

This study used a retrospective cohort design to assess the influence of MARPE on the interdental papilla height of maxillary central incisors. All consecutive patients treated with MARPE between January 2018 and December 2021 at the Department of Orthodontics and Craniofacial Biology of the Radboud University Medical Center were enrolled in this retrospective study in one study cohort. Within this cohort, the patients were followed up prospectively and the analysis of changes in interpapillary height of the central papilla in the maxilla prior to (T0) and one year following MARPE (T1) was performed retrospectively. The study was performed in accordance with the Declaration of Helsinki. Informed consent was obtained from all subjects. All data were anonymized and de-identified prior to analyses, and the study was not subject to the Medical Research Involving Human Subjects Act (WMO) as assessed by the Central Committee on Research Involving Human Subjects (CMO) of the Radboud University Medical Center (IRB no. 2022-15819).

### Participants and study size

The inclusion criteria were non-syndromic patients with transversal maxillary hypoplasia and a minimum age of 16 years. Exclusion criteria were the absence of an approximal contact point between the central incisors before MARPE, and incomplete closure of the central diastema one year after MARPE.

The minimum sample size was calculated at 21 patients based on an alpha of 0.05, a power of 80%, and an assumed effect size of 0.56 (G*Power, version 3.1.9.7., Dusseldorf, Germany). IBM SPSS Statistics for Windows, version 25.0 (IBM Corp., Armonk, NY, USA) was used for data analysis.

### Intervention: MARPE procedure

The Dutch Maxillary Expansion Device (D-MED) (Orthoproof, Nieuwegein, the Netherlands), an individualized digitally designed MARPE appliance for each patient, was used in the present study [13]. It was designed and fabricated based on an intra-oral scan (IOS) (TRIOS 3 scanner, 3Shape, Copenhagen, Denmark) and pre-treatment CBCT (KaVo 3D eXam, KaVo Dental, Biberach, Germany). The IOS was used to determine the shape and location of the device, and CBCT was used to measure the thickness of the palatal bone at the corresponding locations for determining the length of the miniscrews (11 mm or 13 mm; Quattro ®, PSM Medical Solutions; Gunningen, Germany) to ensure bicortical anchorage.

### Treatment procedure

All MARPE appliances were placed by one orthodontist. The D-MED was cemented on both maxillary first molars and served as a surgical guide for miniscrew implantation. After anesthesizing the palatal mucosa at corresponding miniscrew locations, four miniscrews were implanted using a torque of 40 Ncm at 20 rpm, facilitated by using an electric screwdriver (iSD900, PSM Medical Solutions, Gunningen, Germany). If the thickness of the palatal bone was greater than 6 mm, pre-drilling was performed. Frontal intraoral photographs were taken of each patient prior to the start of expansion (T0).

**Fig. 1:**
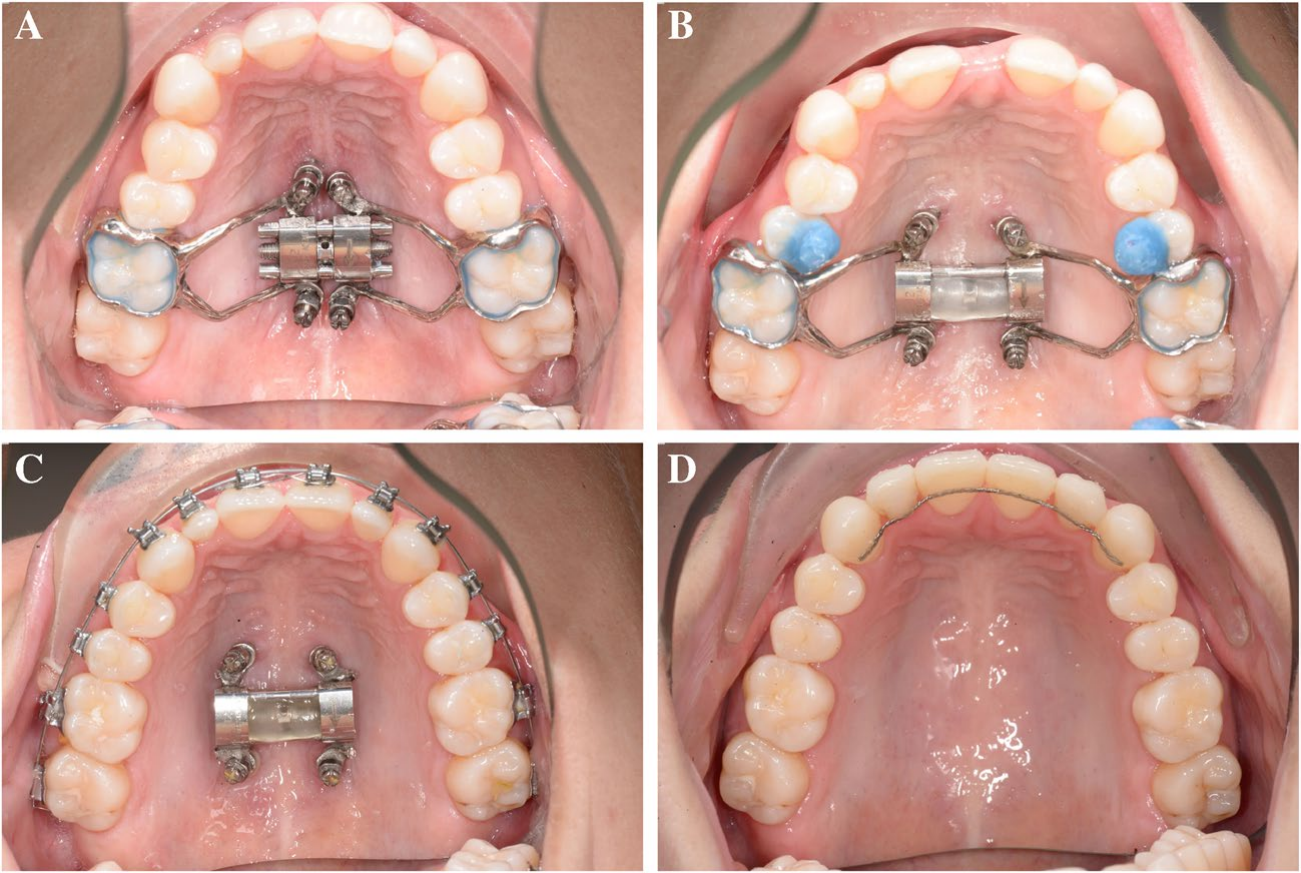
Maxillary occlusal view with D-MED immediately after placement (*T0*) (**A**: top left), immediately after completion of expansion (**B**: top right), 6 months after completion of expansion (**C**: bottom left), and 12 months (*T1*) after completion of expansion (at removal of the MARPE appliance) (**D**: bottom right)

The D-MED was activated immediately after placement (Fig. 1A), and the patient was instructed how to activate the expansion screw and asked to expand once a day, equivalent to 0.25 mm, with weekly follow-up visits at our department, until the expected amount of expansion was achieved. The activation was then stopped and the expansion screw was fixated (Fig. 1B). At 3 months (Fig. 1C) and 12 months (Fig. 1D) after completion of the expansion, the patients attended follow-up visits. After 3 months, the molar bands of the D-MED were removed while the expansion screw was left in place, functioning as a retainer. Simultaneously, orthodontic treatment of the maxilla was initiated. The expansion screw and the miniscrews were removed after 12 months or, in case of orthognathic surgery, 1 month before surgery (T1) when intra-oral photographs were taken and a CBCT was acquired to examine the palatal bone.

### Outcome variables

**Fig. 2:**
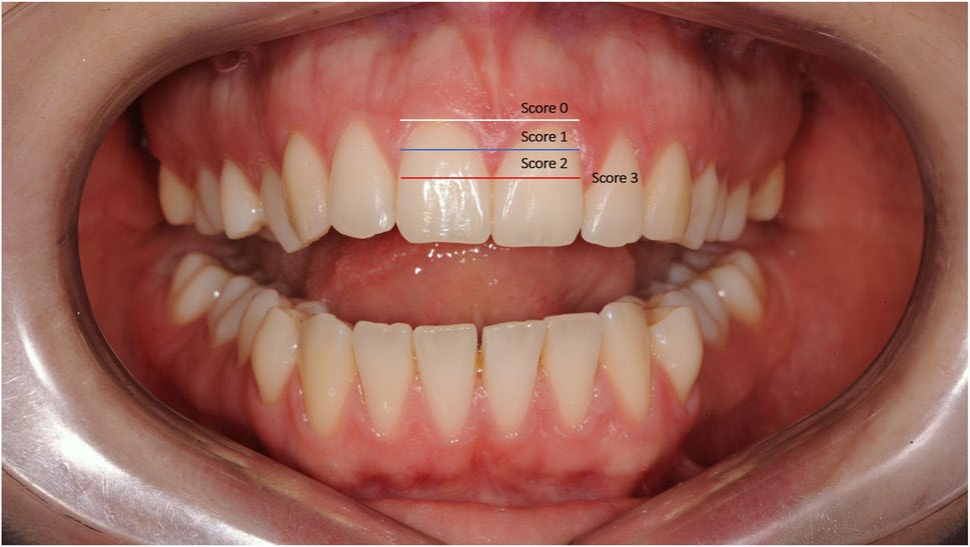
Schematic illustration of the Jemt classification method. The red line went through the approximal contact point of the bilateral central incisors, the white line went through the highest points at the gingival margin and the blue line was placed in the middle between the red and white lines. Score 0 was assigned if the papilla was apical to the white line, score 1 if the papilla was between the blue and white lines, score 2 between the red and blue lines, and score 3 if the papilla was coronal to the red line

The height of the papilla was quantified using Jemt’s classification method based on frontal intraoral photographs taken of each patient at T0 and at T1 [14]. To determine the Jemt score, three parallel lines were drawn in the photographs (Fig. 2): the red line went through the approximal contact point of the bilateral central incisors, the white line went through the highest points at the gingival margin and the blue line was placed in the middle between the red and white lines. Score 0 was assigned if the papilla was apical to the white line, score 1 if the papilla was between the blue and white lines, score 2 between the red and blue lines, and score 3 if the papilla was coronal to the red line.

The primary outcome variable was the difference between the Jemt score (∆Jemt) of the interdental papilla between the maxillary central incisors at T0 and T1. Patients were divided into papilla recession and non-recession groups based on the ∆Jemt.

**Fig. 3:**
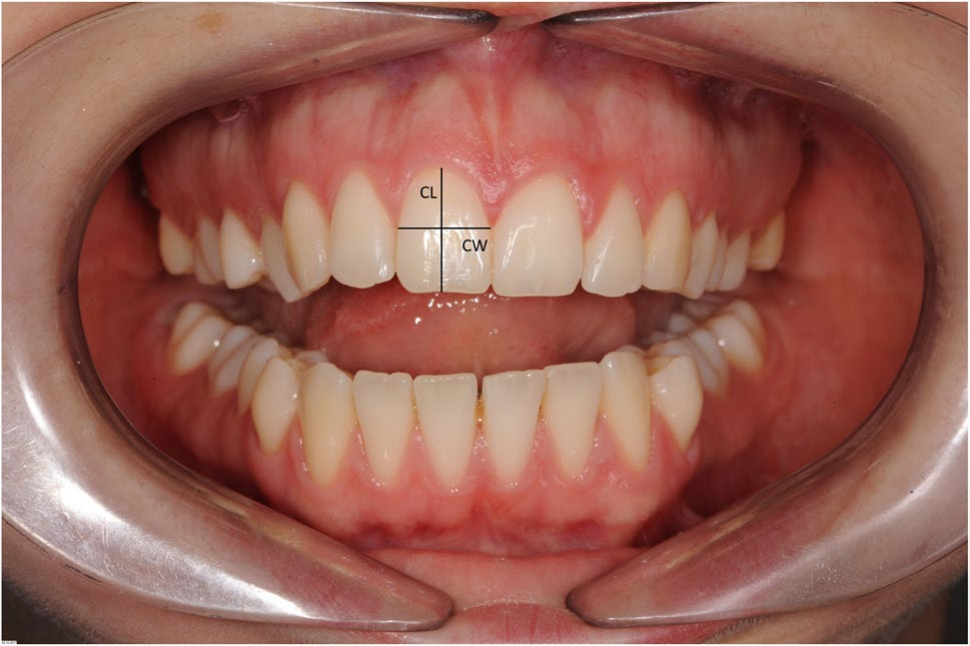
Calculation of tooth morphology as the ratio between crown width (CW)/crown length (CL)

**Fig. 4:**
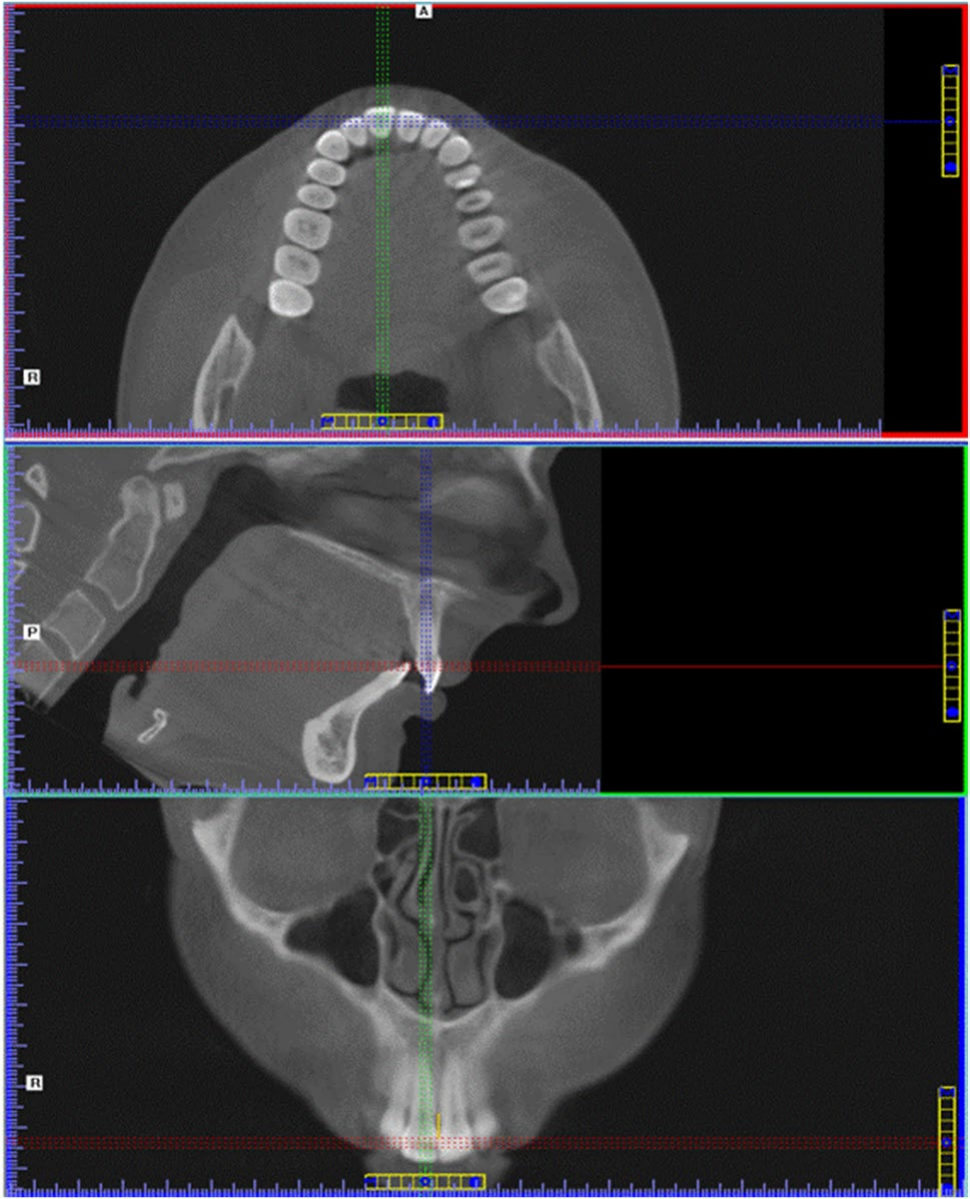
CP-B measurement on CBCT images. **A** (upper): the crown of tooth 11 was centered in the axial plane. **B** (middle): the pulp of tooth 11 was centered in the sagittal plane. **C** (lower): the yellow line gives the CP-B distance in the coronal plane

In addition to the Jemt classification, the following variables were recorded:

- Age (years), gender (male/female), Angle classification (I/II/III), MARPE treatment duration (months)
- Pretreatment overlap of maxillary central incisors (yes/no)
- Crown morphology assessed at T0 by using the ratio between crown width and crown length (W: L) (Fig. 3). Crown width (CW) was defined as the distance between the approximal surfaces of the adjacent teeth, and crown length (CL) was defined as the distance between the incisal edge and the gingival margin [15].
- The distance between the bone crest and approximal contact point of the central incisors (CP-B) was measured on the CBCT scans at T0 and T1 (mm). The long axis of the right maxillary central incisor was aligned with the vertical plane. CP-B was defined as the distance from the bone crest to the contact point between the maxillary central incisors in the coronal plane (Fig. 4) [3], and Δ CP-B was defined as the change in distance of CP-B from T0 to T1.
- Maximal central diastema (MCD) defined as the clinical maximal diastema width (mm) between the maxillary central incisors at the end the activation phase with MARPE.
- Midpalatal suture maturation stage (MSM) determined on CBCT at T0 according to the classification of F. Angelieri et al. [16].

### Statistical methods

All measurements were performed twice by one observer (HC) with an interval of 2 weeks to test for intra-observer reliability. Descriptive statistics were used to describe the demographics of the study population. The reliability of measurements was determined using kappa statistics for categorical variables and the intraclass correlation coefficient (ICC) for continuous variables. The occurrence of each Jemt score at T0 and T1 was computed. Fisher’s exact test and independent samples *t*-test were used to compare the categorical and continuous variables between the papilla recession and non-recession groups, respectively.

## Results

Thirty-one patients underwent MARPE between January 2018 and December 2021. Five patients were excluded due to the absence of a contact point between the central incisors before MARPE, and four patients were excluded because of incomplete closure of the central diastema one year following MARPE. Twenty-two participants were finally enrolled.

The study population consisted of 2 male and 20 female, with a mean age of 27.3 ± 8.8 years (Table 1). The mean interval between T0 and T1 was 17.5 ± 4.3 months. Both the Kappa values of the categorical variables (overlapping: 0.879; Jemt T0: 0.91; Jemt T1: 0.91; MSM 0.96) and the ICC for the continuous variables (CP-B T0: 0.93; CP-B T1: 0.90; W: L: 0.97) were high, indicating an excellent intra-observer reliability.

**Table 1:** Characteristics of the study population

Variable		Total (%)
Age at start of expansion (years)	Mean ± SD	27.3 ± 8.8
Gender	Male	2 (9%)
	Female	20 (91%)
Angle classification	Class I	4 (18%)
	Class II/1	13 (59.1%)
	Class II/2	3 (14%)
	Class III	2 (9%)
Midpalatal suture maturation	Stage A	1 (4.5%)
	Stage B	1 (4.5%)
	Stage C	4 (18.2%)
	Stage D	8 (36.4%)
	Stage E	8 (36.4%)
Overlapping	Overlapping	5 (23%)
	No overlapping	17 (77%)
Treatment duration (months)	Mean ± SD	17.50 ± 4.27
Maximal central diastema (mm)	Mean ± SD	4.20 ± 1.86
Crown morphology (width/length)	Mean ± SD	0.79 ± 0.07
Δ CP-B (mm)	Mean ± SD	0.86 ± 0.52

*SD* standard deviation, *CP-B* distance between the bone crest and approximal contact point of the central incisors

**Fig. 5:**
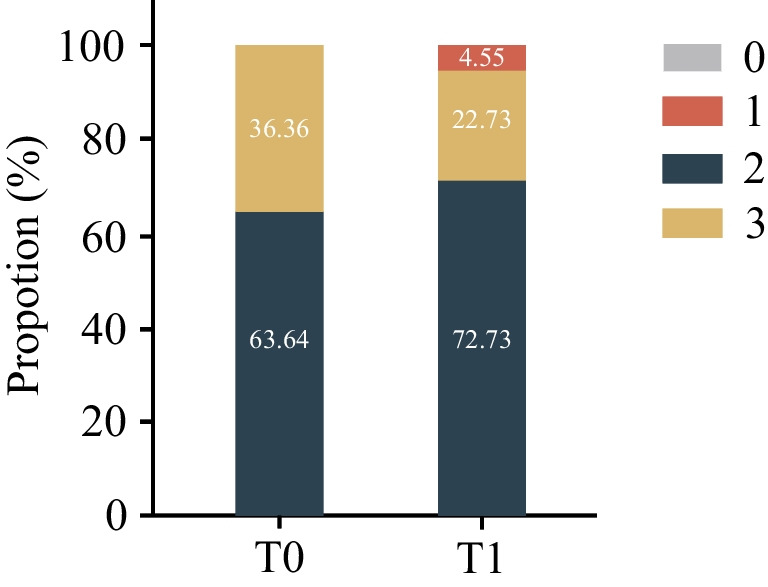
Distribution of Jemt score at T0 and T1. There were no patients with a Jemt score of 0 at T0 and T1, and no patients with a Jemt score of 1 at T0

Overall, there was a decrease in Jemt score from a mean of 2.36 (SD: 0.49) at T0 to 2.18 (SD: 0.50) at T1 (*p* = 0.04). Four patients (18%) demonstrated a decrease in Jemt score between T0 and T1 (Fig. 5). All patients with papilla recession had overlapping central incisors at the start of treatment (Table 2). The odds ratio for the occurrence of papilla recession in the case of incisal overlap was 17.2. The mean maximal central diastema in the papilla recession group (2.25 ± 2.06 mm) was significantly smaller than in the non-recession group (4.64 ± 1.55 mm; *p* = 0.02). The patients in the papilla recession group experienced a larger increase in CP-B compared to the non-recession group, 1.83 mm vs 0.64 mm (*p* = 0.02), respectively. The W/L ratio was significantly smaller in the recession group compared to the non-recession group (Table 3). No differences in Angle classification, MSM, age and treatment duration were present among patients with and without papilla recession.

**Table 2:** Comparison of the categorical variables between papilla recession and non-recession groups

Variable		Recession (*n*)	Non-recession (*n*)	*p* value
Gender	Male	0	2	0.48
	Female	4	16	
Angle classification	Class I	1	3	0.51
	Class II/1	3	10	
	Class II/2	0	3	
	Class III	0	2	
MSM	Stage A	0	1	0.14
	Stage B	1	0	
	Stage C	1	3	
	Stage D	0	8	
	Stage E	2	6	
Overlapping	Overlapping	4	1	< 0.01*
	No overlapping	0	17	

*MSM* midpalatal suture maturation

*Statistically significant (Fisher’s exact test)

**Table 3:** Comparison of the continuous variables between papilla recession and non-recession groups

Variable	Recession Mean (SD)	Non-recession Mean (SD)	Mean difference 95%CI [lower; upper]	*p* value
Age (years)	32.39 (12.33)	26.20 (7.79)	− 6.19 [− 16.13;3.76]	0.21
Treatment duration (months)	15.75(4.99)	17.89(4.16)	2.14 [− 2.81;7.09]	0.38
MCD (mm)	2.25 (2.06)	4.64 (1.55)	2.39 [0.50;4.28]	0.02*
W/L ratio	0.71 (0.06)	0.82 (0.06)	0.11 [0.04;0.17]	< 0.01*
ΔCP-B (mm)	1.83 (0.48)	0.64 (0.17)	− 1.18 [− 1.92; − 0.43]	0.02*

*CI* confidence interval of the difference, *MCD* maximal central diastema

*Significant (independent *t*-test)

## Discussion

In the present study, 4 of 22 patients (18%) exhibited mild recession of the interdental papilla between the maxillary central incisors following MARPE treatment. The presence of an overlap of central incisors before treatment, a smaller central diastema at the end of the activation phase of MARPE, an increased distance between the bone crest and approximal contact point following MARPE, and a smaller width-to-length ratio of the central incisors prior to treatment being identified as factors that were associated with papilla recession.

Papilla recession in the esthetic zone can compromise a beautiful smile due to the formation of “black triangles” [4]. Papilla recession not only affects esthetics but may also cause food impaction, deteriorating periodontal health. Treatment of papilla recession is complex, often not predictable in the long term, and mostly requires an interdisciplinary approach, involving combined periodontal, orthodontic and restorative treatment [17, 18]. Orthodontic treatment, and maxillary expansion in particular, may cause papilla recession [19]. With MARPE, the maxillary central incisors are gradually moving apart during the activation phase, resulting in a temporary diastema and an open gingival embrasure. Although the diastema is closed following the retention phase and orthodontic treatment, changes in the height of the papilla may be a concern for the orthodontist, as papilla recession occurred in 4 of 22 (18%) patients. Overall, the Jemt score showed a decreasing trend from T0 (2.36 ± 0.49) to T1(2.18 ± 0.50) (*p* = 0.04). A mean Jemt score of 2.18 after MARPE implied that in an average patient, half or more of the preoperative papilla height was still present following MARPE. The amount of papilla recession following MARPE was limited, and may not influence the overall aesthetic of the smile line. However, the presence of incisal overlap of the central incisors, MCD, an increase in the CP-B and a narrow crown morphology were associated with a higher risk of papilla recession.

In comparison, the incidence of gingival black triangles following orthodontic treatment with fixed appliances ranged from 38 to 58% [2]. Our results showed that papilla recession occurred following MARPE in 18% of the patients, which was relatively low. The incidence of papilla recession following MARPE was also lower than following SARME (32%) as reported in a recent study [3]. As the characteristics of the study population from both studies were similar, except for treatment duration, it seems that the absence of a surgical osseous split between the central incisors may be associated with a lower incidence of papilla recession.

Previous studies showed that the height of CP-B was associated with the integrity of gingival tissue between the interproximal contact [20, 21]. Chen et al. demonstrated in their study that the papilla was always present when the distance between the crestal bone and approximal contact point of the central incisors was 5 mm or less. When the distance was 7 mm or more, the papilla was usually missing [20]. The papilla height was dependent on the height and underlying contour of the bone crest, i.e. the higher the crestal bone, the higher the gingival papilla [22]. The results of the present study showed that a significant reduction in CP-B occurred as a result of MARPE treatment, similarly to SARME. In both MARPE and SARME, the crestal bone between the central incisors did not return to its pretreatment level following the expansion phase, despite closure of the diastema. As no direct osteotomy was performed in the crestal bone area among MARPE patients, the increase of CP-B following MARPE was less than following SARME. This could explain the lower incidence of papilla recession among MARPE patients.

An et al. stated that overlap between the maxillary central incisors before treatment was associated with open gingival embrasures (black triangles) after orthodontic treatment. With each millimeter of initial anteroposterior overlap, the occurrence of open gingival embrasures increased by 2.2 [23]. The incidence of open gingival embrasure spaces after orthodontic alignment of overlapped and crowded maxillary central incisors was 40% [24]. In the present study, all patients with papilla recession had at least some degree of overlapping maxillary central incisors, highlighting the relationship between overlap of central incisors and papilla recession. During MARPE, the approximal contact points of the central incisors are separated. Following treatment with fixed appliances and closure of the central diastema, a new interdental contact point is established between the central incisors. However, the interdental contact point of the overlapped incisors may be positioned more towards the incisal edge, which may subsequently lead to an increase of CP-B, resulting in papilla recession. When aligning the incisors with fixed appliances after the expansion phase, the incisors may need to move in the antero-posterior direction and undergo rotational movements [2, 23, 25]. The increased amount of movement may lead to more bone loss, thereby increasing CP-B and leading to subsequent loss of the papilla height, which could also explain papilla recession in patients with overlapping incisors.

The height of the interdental papilla was also reported to be associated with the crown width to length ratio. When W/L is larger than 0.87, less papilla recession could be expected [26]. In the present study, the W/L ratio in the non-recession group was significantly larger than that in the recession group, which was in line with the previous findings. In other words, central incisors that were more square-shaped and less rectangular were more likely to have an adequate papilla. Incisors that are more square-shaped tend to have more interproximal contact surface and have a contact point that is located closer to the papilla [27]. The CP-B increase following MARPE would, thus, be less in incisors with a larger W:L ratio.

In the present study, the mean MCD was significantly greater in the non-recession group than in the recession group. However, the multiple regression analysis did not identify MCD as a prognostic factor for papilla recession. These findings suggest that MCD was not a predictor of papilla recession.

We also found that age was not significantly associated with papilla recession. On the other hand, previous studies described that an increased age is associated with papilla recession [4, 28]. This discrepancy may be the result of the size of the study population in this study. A larger study population is required to investigate whether older patients who underwent MARPE may have a higher incidence of papilla recession compared to younger patients.

A limitation of this study is that although the sample size was sufficient to obtain a sufficient power in testing for differences in Jemt score between T0 and T1, the sample size and strong collinearities among the data precluded multivariable linear or logistic regression analyses involving multiple independent factors such as W:L ratio and incisal overlapping, which aimed to estimate the individual effects of the independent variables on papilla recession. Some factors that are associated with papilla recession, such as smoking, tooth brushing technique, gingival inflammation, gingival thickness, and width of the crestal alveolar bone [29] between the central incisors were also not quantified. A much larger study population would be required to investigate the potential risk factors for papilla recession following MARPE in a future clinical study.

## Conclusion

Following MARPE treatment, 18% of patients showed a mild recession of papilla height between the maxillary central incisors. Within the limitations of the present study, overlap of the central incisors before treatment, a small central diastema immediately after expansion, an increase in distance between the bone crest and approximal contact point and a smaller width to length ratio of the central incisors were associated with papilla recession. Furthermore, overlapping and a smaller width to length ratio of the maxillary central incisors were predictors for interdental papilla recession. Clinicians should inform patients who are to undergo MARPE about the risk of papilla recession.
